# Quality assurance for Chinese herbal formulae: standardization of IBS-20, a 20-herb preparation

**DOI:** 10.1186/1749-8546-5-8

**Published:** 2010-02-22

**Authors:** Siu-Po Ip, Ming Zhao, Yanfang Xian, Mengli Chen, Yuying Zong, Yung-Wui Tjong, Sam-Hip Tsai, Joseph JY Sung, Alan Bensoussan, Brian Berman, Harry HS Fong, Chun-Tao Che

**Affiliations:** 1 School of Chinese Medicine, Chinese University of Hong Kong, Hong Kong SAR, China; 2 Department of Medicine and Therapeutics, Chinese University of Hong Kong, Hong Kong SAR, China; 3 Centre for Complementary Medicine Research, University of Western Sydney, Australia; 4 Center for Integrative Medicine, University of Maryland School of Medicine, Baltimore, Maryland, USA; 5 Department of Medicinal Chemistry and Pharmacognosy, College of Pharmacy, University of Illinois at Chicago, Chicago, USA

## Abstract

**Background:**

The employment of well characterized test samples prepared from authenticated, high quality medicinal plant materials is key to reproducible herbal research. The present study aims to demonstrate a quality assurance program covering the acquisition, botanical validation, chemical standardization and good manufacturing practices (GMP) production of IBS-20, a 20-herb Chinese herbal formula under study as a potential agent for the treatment of irritable bowel syndrome.

**Methods:**

Purity and contaminant tests for the presence of toxic metals, pesticide residues, mycotoxins and microorganisms were performed. Qualitative chemical fingerprint analysis and quantitation of marker compounds of the herbs, as well as that of the IBS-20 formula was carried out with high-performance liquid chromatography (HPLC). Extraction and manufacture of the 20-herb formula were carried out under GMP. Chemical standardization was performed with liquid chromatography-mass spectrometry (LC-MS) analysis. Stability of the formula was monitored with HPLC in real time.

**Results:**

Quality component herbs, purchased from a GMP supplier were botanically and chemically authenticated and quantitative HPLC profiles (fingerprints) of each component herb and of the composite formula were established. An aqueous extract of the mixture of the 20 herbs was prepared and formulated into IBS-20, which was chemically standardized by LC-MS, with 20 chemical compounds serving as reference markers. The stability of the formula was monitored and shown to be stable at room temperature.

**Conclusion:**

A quality assurance program has been developed for the preparation of a standardized 20-herb formulation for use in the clinical studies for the treatment of irritable bowel syndrome (IBS). The procedures developed in the present study will serve as a protocol for other poly-herbal Chinese medicine studies.

## Background

Herbal medicines, whether in the form of single herb phytomedicine or multiple herb mixtures, are popular around the world. However, evidence of efficacy and safety has not been well documented [1]. Lack of effective quality assurance affects the efficacy and safety assessment of herbal products [1-7]. For valid pharmacological or clinical efficacy evaluations, a standardized single batch clinical formulation should be employed. As part of a research project to evaluate the therapeutic potential of a 20-herb Chinese medicine formula (the IBS-20 formula) for treating irritable bowel syndrome (IBS), we have developed and tested a quality assurance program for the production of the multi-herb preparation. IBS affects 10-20% of the global population [8] and it has not been successfully treated with conventional medications such as bulking, smooth muscle relaxant, prokinetic and psychotropic agents, nor loperamide and peppermint oil [9].

We are presently conducting a clinical study of a 20-herb Chinese medicine formula (IBS-20) to determine its efficacy potential in the treatment of this disorder. To insure the validity and reproducible results in conducting this study, we established a robust quality assurance (QA)/quality control (QC) program. The present paper describes the methods employed, covering all aspects of the production of IBS-20 from source material acquisition, botanical validation, chemical standardization, extraction and formulation. The protocols established in this study may be used as a model for the quality assurance of other herbal products.

## Methods

### Plant materials

The component herbs in the formula are as follows: *Pogostemon cablin *(herb) (4.5%, w/w), *Angelica dahurica *(root) (2%), *Artemisia scoparia *(herb) (13%), *Atractylodes macrocephala *(rhizome)(9%), *Aucklandia lappa *(root) (3%), *Bupleurum chinense *(root) (4.5%), *Citrus reticulate *(fruit peel) (3%), *Codonopsis pilosula *(root) (7%), *Coix lacryma-jobi *(seed) (7%), *Coptis chinensis *(rhizome) (3%), *Fraxinus rhynchophylla *(bark) (4.5%), *Glycyrrhiza uralensis *(root) (4.5%), *Magnolia officinalis *(bark) (4.5%), *Paeonia lactiflora *(root)(3%), *Plantago asiatica *(seed) (4.5%), *Phellodendron amurense *(bark) (4.5%), *Poria cocos *(fruiting body) (4.5%), *Saposhnikovia divaricata *(root) (3%), *Schisandra chinensis *(fruit) (7%) and *Zingiber officinale *(rhizome) (4.5%). All 20 herbs were acquired in the prescribed proportions (% w/w) from Zhixin Chinese Pharmaceutical Co. Ltd. (Guangzhou, China). The aggregate weight of the 20 herbs was 400 kg (Additional File 1). Voucher samples (#IBS-01 to IBS-20) were deposited at the herbarium of the School of Chinese Medicine, Chinese University of Hong Kong (Hong Kong SAR, China). The individual bulk herb samples were stored in air-tight containers kept in air-conditioned environment until use. The herbs were identified in both Chinese and botanical (Latin binomial) names. When two or more species share the same Chinese name, only one species was selected for chemical and biological/clinical studies.

### Botanical authentication

All 20 herbs were authenticated macroscopically and microscopically. Macroscopic examinations included measurements of appearance, size, shape, color, texture, odor, taste, fracture and other characteristics of a herb according to pharmacopoeias [10-13]. Microscopic examinations determined characteristic elements of each herb in both tissue and powder forms. In cross sectional examination, herbal material was softened by immersion in water, alcohol or glycerin prior to sectioning. Sliced tissue, prepared with a microtome, was mounted on a glass microscope slide and clarified with chloral hydrate, lactochloral and/or sodium hypochlorite, followed by phloroglucinol, potassium iodide or Sudan Red. In powder analysis, each herbal material was pulverized to 65-mesh in size, mounted on a microscope slide, cleared with chloral hydrate, lactochloral and/or sodium hypochlorite, and then examined for the presence, size, shape and numbers of characteristic elements and inclusions such as vessels, calcium crystals, crystalline fibers, stone cells and starch grains. The examination protocols followed the World Health Organization (WHO) Quality Control Methods for Medicinal Plant Materials [14], the Pharmacopoeia of the People's Republic of China (CP) [10] and the Hong Kong Chinese Materia Medica Standards (HKCMMS) [11-13]. The recorded macroscopic and microscopic data for each herb were verified against those in the CP and/or the HKCMMS, coupled with visual comparison with available reference samples.

### Reference marker compounds and reagents

Reference marker compounds for qualitative and quantitative high performance liquid chromatography (HPLC) were obtained from the National Institute for the Control of Pharmaceutical and Biological Products (Beijing, China) and further validated by mass spectrometry (MS) and nuclear magnetic resonance spectroscopy (NMR) and purity (>98%) analysis with HPLC and/or liquid chromatography-mass spectrometry (LC-MS). Chemicals and general solvents were of reagent grade and HPLC solvents were of HPLC grade (BDH, United Kingdom).

### Purity and contaminant determination

Purity rubric tests, including foreign matters, total ash, acid-insoluble ash, water and extractive contents, were carried out according to the CP or HKCMMS [10-13]. Determination of heavy metals (arsenic, cadmium, mercury, and lead), pesticides, microbials and microbial toxin (aflatoxin) was carried out according to the HKCMMS [11-13]. Briefly, for heavy metal analysis, the herbal matrix was dissolved by microwave-assisted acid digestion, and the presence and quantity of mercury, lead, arsenic, and/or cadmium, if any, were determined by inductively coupled plasma-mass spectrometry (ICP-MS). Pesticide residues (e.g., aldrin, dieldrin, chlordane, dichlorodiphenyltrichloroethane, endrin, heptachlor, hexachlorobenzene, hexachlorocyclohexane isomers, lindane and quintozene) were quantitatively determined with gas chromatography (GS). Mycotoxins (aflatoxins B_1_, B_2_, G_1 _and G_2_) were detected as previously described [11-13,15]. Microorganism examinations included total bacteria, mould and yeast, *Escherichia coli *and *Salmonella *counts as described in the CP [10].

### Chemical standardization

*Chromatographic fingerprint analysis *HPLC fingerprinting with one or more reference markers was carried out according to the HKCMMS and/or CP [10-13]. As an example, the procedure used for *Rhizoma Coptidis *(Huanglian) is described here. The herb was ground to powder, extracted in MeOH by ultrasonication for 30 minutes and filtered. The chromatographic system consisted of an Agilent 1100 HPLC system (Agilent Technologies, USA) equipped with a secondary pump, a diode-array detector, an autosampler, and a column compartment, an Alltech Alltima C18 column (4.6 × 250 mm) (Alltech, USA) packed with 5 μm diameter particles and an Alltech Alltima guard column (7.5 × 4.6 mm, 5 μm) (Alltech, USA); solvent system: 0.1% trifluoroacetic acid (%, v/v) (A) and acetonitrile (%, v/v) (B) with a linear gradient elution, 0% B-50% B at 0-48 minutes, 50% B-100% B at 48-55 minutes, 100% B was held for five minutes; flow rate: 1.0 ml/min; detection: 346 nm; reference marker: berberine. Information of the reference marker compounds for each herb is available in Additional File 1.

#### Quantitative analysis

Quantitative determination of selected marker compound(s) in each herb was performed with HPLC analysis. As an example, the quantitative analysis of *Cortex Magnoliae Officinalis *(Houpo) is described here. Preparation of the herb and the HPLC setup were the same as described above. The mobile phase contained 0.4% formic acid and acetonitrile (35:65); flow rate: 1 ml/min; detection: 294 nm. Information of the reference marker compounds for each herb is available in Additional File 1.

#### Inter-laboratory methods validation

Inter-laboratory validation of fingerprint and quantitative HPLC analytical protocols were carried out in laboratories at the Chinese University of Hong Kong and the University of Western Sydney prior to use. Both laboratories followed the identical experimental protocols and the results were critically compared.

### Production of herbal extracts and the IBS-20 formula

The 20 dried herbs were individually reduced in size by milling or slicing and mixed in the prescribed proportion (% w/w), followed by extraction with water under GMP at the Hong Kong Institute of Biotechnology (Hong Kong Special Administrative Region, China). Briefly, the herb mixture (400 kg) was decocted with 10-fold (w/v) of boiling distilled water for 60 minutes, cooled and collected. After fresh boiling water was added, the mixture was decocted for a second time. The cooled extracts were pooled, filtered, concentrated and spray dried to obtain a powder (34% yield w/w based on raw herbs). An aliquot was set aside for chemical and pre-clinical biological studies. The remaining powdered extract was then formulated with water-soluble starch (excipient) in a ratio of 1:1 into the clinical product in the form of an aluminum foil packed sachet.

### Chemical standardization of the IBS-20 formula

Chemical standardization of the clinical herbal extract with selected reference markers was performed with HPLC coupled with diode array detection and atmospheric pressure chemical ionization mass spectrometry (HPLC-DAD-APCIMS) analysis. Briefly, the powdered extract was sonicated in MeOH for 30 minutes and filtered through a cellulose syringe filter. An aliquot (10 μl) of filtrate was injected into an Agilent 1100 HPLC system equipped with an Alltech Alltima C18 column (4.6 × 250 mm, 5 μm diameter). The mobile phase consisted of 0.1% trifluoroacetic acid in water (v/v) (A) and acetonitrile (B) with the gradient elution conditions as follows: 12% B-13% B at 0-10 minutes, 13% B - 16% B at 10-40 minutes, 16% B-36.4% B at 40-67 minutes, 36.4% B-60% B at 67-100 minutes, from 100-120 minutes a gradient was applied to 100% B and was held for five minutes, followed by a 10-minute equilibration period at 12% B; flow rate: 1.5 ml/min; temperature: 27.5°C (constant). For detection, an Agilent 1100 series LC/MSD trap (Agilent Technologies, USA) was connected to the HPLC system via an APCIMS interface. Ultra-high purity helium was used as the collision gas and high purity nitrogen as the nebulizing gas. The optimized parameters in the positive ionization mode were as follows: nebulizer gas pressure: 50 psi; dry gas flow: 5.0 l/min; dry temperature: 350°C; vaporizer temperature: 400°C; full-scan MS analysis in the range of *m/z *100-2200. The reference marker compounds present in the sample were identified by retention time, MS fragmentation and UV spectra.

### Stability monitoring

The stability of the chemically standardized herbal formula was monitored in real time over a period of at least one year. The HPLC fingerprint profiles as well as the quantitative content of eight selected major marker compounds in the clinical formula were measured on days 0, 2, 3, 7, 14, 30, 60, 90, 180, 360, 450 post-production with HPLC and LC-MS respectively.

## Results and discussion

**Figure 1:**
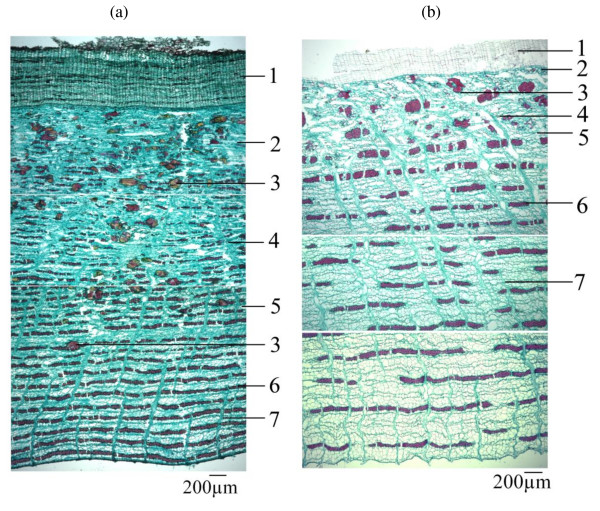
**Microscopic features of cross section**. (a) *Phellodendron chinensis *bark. (b). *Phellodendron amurense *bark 1: Cork; 2: Cortex; 3: Stone cells; 4: Prisms of calcium oxalate; 5: Phloem; 6: Phloem fibres and crystal fibres; 7: Phloem rays.

**Figure 2:**
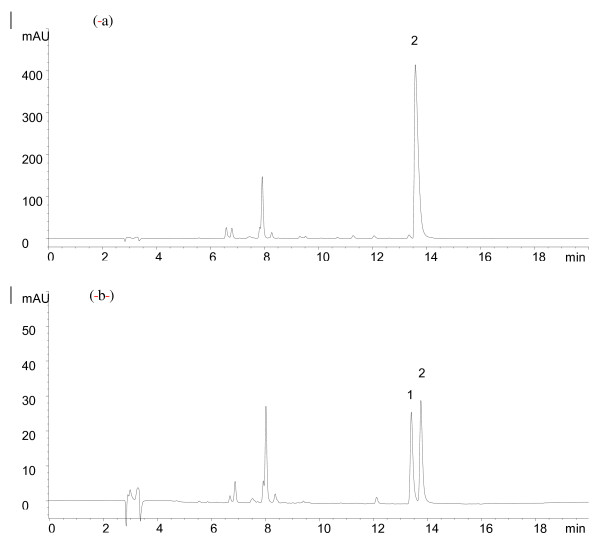
**HPLC chromatograms**. (a). *Phellodendron chinensis *bark. (b) *Phellodendron amurense *bark 1: palmatine; 2: berberine.

During the selection of herbs, we paid special attention to the cases where the Chinese names correspond to more than one species. For example, *Cortex Phellodendri *refers to the bark of either *Phellodendron amurense *or *Phellodendron chinense *which are similar in macroscopic appearance and used interchangeably in Chinese medicine. Our studies in support of the HKCMMS [11] revealed significant differences in microscopic and chemical profiles of the two species using thin-layer chromatographic (TLC) and HPLC fingerprints as well as differences in contents using quantitative HPLC analysis. Microscopically, the cortex of *Phellodendron chinense *is broader than that of *Phellodendron amurense *(Figure 1), while the stone cells are more abundant and scattered in the outer layer of phloem of the former species (Figure 1a) but are sparsely scattered in the cortex of the latter (Figure 1b). Chemically, the marker compound profiles also differ significantly (Figure 2). In *Phellodendron chinense *(Figure 2a), berberine predominates while palmatine is not discernible. On the other hand, both berberine and palmatine are present and appear to be similar in concentration in the cortex of *Phellodendron amurense *(Figure 2b). Thus, to ensure chemically and biologically reproducible batches, we decided to use the cortex of *Phellodendron amurense *in our preparation.

Purity rubric tests indicated that the herbs met the limits established by the CP and/or HKCMMS [10-13] (in the cases where regulatory standards are available) (Table 1). Tests for contaminants showed that none of the 20 herbs exceeded the standards established by the CP and/or HKCMMS [10-13] (Table 2).

**Table 1:** Regulatory standards and experimental results of purity and contaminant tests

			Ash						Extractives			
						
Pharmaceutical name	Foreign matters		Total ash		Acid-insoluble Ash		Water content		Water-soluble extractives		Ethanol-soluble extractives	
*Radix Angelicae Dahuricae*	--	(<1.0%)	6.0%^a^	(4.1%)	1.5%^a^	(<1.0%)	14%^a^	(10%)	--	(27%)	15%^a^	(17%)
*Herba Artemisiae Scopariae*	--	(<1.0%)	4.0%^a^	(2.9%)	2.0%^a^	(<1.0%)	15%^a^	(9.7%)	--	(25%)	--	(17%)
*Rhizoma Atractylodis Macrocephalae*	--	(<1.0%)	5.0%^a^	(3.7%)	1.0%^a^	<1.0%)	--	(10%)	--	(67%)	--	(12%)
*Radix Aucklandiae*	2.0%^b^	(<1.0%)	4.5%^b^	(3.2%)	1.0%^b^	(<1.0%)	14%^b^	(10%)	65%^b^	(68%)	15%^b^	(27%)
*Radix Bupleuri*	2.0%^b^	(<1.0%)	7.7%^b^	(1.0%)	3.5%^b^	(2.8%)	5.0%^b^	(4.4%)	12%^b^	(22%)	11%^b^	(17%)
*Pericarpium Citri Reticulatae*	--	(<1.0%)	--	(4.7%)	--	(1.4%)	13%^a^	(11%)	--	(39%)	--	(39%)
*Radix Codonopsis*	1.0%^b^	(<1.0%)	6.0%^b^	(4.1%)	2.5%^b^	(<1.0%)	12%^b^	(10%)	41%^b^	(60%)	21%^b^	(52%)
*Semen Coicis*	2.0%^a^	(<1.0%)	3.0%^a^	(2.3%)	--	(<1.0%)	15%^a^	(9.9%)	--	(6.0%)	5.5%^a^	(6.0%)
*Rhizoma Coptidis*	2.0%^b^	(<1.0%)	5.0%^b^	(2.5%)	2.5%^b^	(<1.0%)	12%^b^	(7.5%)	17%^b^	(23%)	14%^b^	(19%)
*Cortex Fraxini*	--	(<1.0%)	8.0%^a^	(4.7%)	--	(1.5%)	7.0%^a^	(6.5%)	--	(8.0%)	8.0%^a^	(8.7%)
*Radix et Rhizoma Glycyrrhizae Praeparata cum Melle*	--	(<1.0%)	5.0%^a^	(3.1%)	1.0%^a^	(<1.0%)	10%^a^	(8.1%)	--	(47%)	--	(43%)
*Cortex Magnoliae Officinalis*	1.0%^b^	(<1.0%)	8.0%^b^	(4.50%)	3.5%^b^	(1.6%)	12%^b^	(8.3%)	3.0%^b^	(8.0%)	5.0%^b^	(9.0%)
*Radix Paeoniae Alba*	1.0%^b^	(<1.0%)	4.0%^b^	(2.1%)	1.0%^b^	(<1.0%)	14%^b^	(7.1%)	21%^b^	(22%)	16%^b^	(17%)
*Semen Plantaginis*	--	(<1.0%)	6.0%^a^	(3.2%)	2.0%^a^	(<1.0%)	12%^a^	(10%)	--	(12%)	--	(3.3%)
*Cortex Phellodendri Amurensis*	1.0%^b^	(<1.0%)	8.5%^b^	(7.3%)	1.0%^b^	(<1.0%)	11%^b^	(8.7%)	9.0%^b^	(17%)	12%^b^	(16%)
*Herba Pogostemonis*	2.0%^a^	(<1.0%)	11%^a^	(8.6%)	4.0%^a^	(2.3%)	14%^a^	(9.1%)	--	(14%)	2.5%^a^	(10%)
*Poria*	--	(<1.0%)	4.0%^a^	(2.4%)	2.0%^a^	(<1.0%)	15%^a^	(9.5%)	--	(2.0%)	--	(2.6%)
*Radix Saposhnikoviae*	2.0%^b^	(<1.0%)	7.0%^b^	(5.1%)	2.5%^b^	(1.8%)	13%^b^	(7.7%)	22%^b^	(27%)	19%^b^	(25%)
*Fructus Schisandrae Chinensis*	1.0%^a^	(<1.0%)	--	(4.5%)	--	(1.4%)	--	(12%)	--	(30%)	--	(31%)
*Rhizoma Zingiberis Praeparatum*	--	(<1.0%)	7.0%^a^	(4.6%)	--	(<1.0%)	--	(5.8%)	--	(13%)	--	(7.8%)

Note: Data in parentheses are experimental results of the samples.

^a^ Limit required by the Pharmacopoeia of the People's Republic of China (2005 edition)

^b^ Limit required by the Hong Kong Chinese Materia Medica Standards

**Table 2:** Limits and experimental results of toxic contaminant tests

Test	Limit (maximum)	Herbal formula
**Heavy metals:**		
Arsenic (As)	2.0 mg/kg^b^	0.30 mg/kg
Cadmium (Cd)	0.3 mg/kg^b^	0.13 mg/kg
Mercury (Hg)	0.2 mg/kg^b^	Not detectable
Lead (Pb)	5.0 mg/kg^b^	0.34 mg/kg
**Pesticide residues:**		
Aldrin and dieldrin (sum of)	0.05 mg/kg^b^	Not detectable
Chlordane (sum of *cis*-, *trans*- and oxychlordane)	0.05 mg/kg^b^	Not detectable
DDT (sum of p,p'-DDT, o,p'-DDT, p,p'-DDE and p,p'-TDE)	1.0 mg/kg^b^	Not detectable
Endrin	0.05 mg/kg^b^	Not detectable
Heptachlor (sum of heptachlor and heptachlor epoxide)	0.05 mg/kg^b^	Not detectable
Hexachlorobenzene	0.1 mg/kg^b^	Not detectable
Hexachlorocyclohexane isomers (α-, β- and δ- hexachlorocyclohexane)	0.3 mg/kg^b^	Not detectable
Lindane (γ-hexachlorocyclohexane)	0.6 mg/kg^b^	Not detectable
Quintozene (sum of quintozene, pentachloroaniline and methyl pentachlorophenyl sulphide)	1.0 mg/kg^b^	Not detectable
**Mycotoxins:**		
Aflatoxin B1	5 μg/kg^b^	Not detectable
Sum of aflatoxins B1, B2, G1 and G2	10 μg/kg^b^	Not detectable
**Microbiological:**		
Total plate counts	1000 colony/g^a^	< 10 colony/g
Yeast and mould	100 colony/g^a^	< 10 colony/g
*Escherichia coli*	Absent^a^	Absent
*Salmonella *species	Absent	Absent

^a ^Limit required by the Pharmacopoeia of the People's Republic of China (2005 edition)

^b ^Limit required by the Hong Kong Chinese Materia Medica Standards

**Figure 3:**
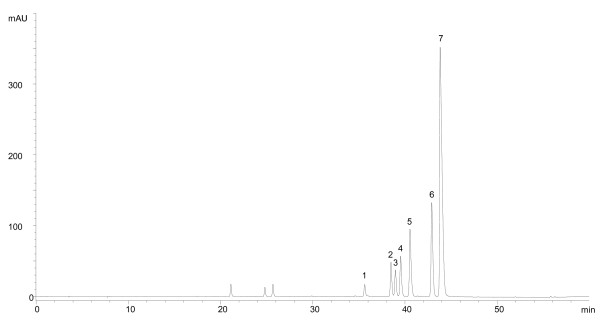
**Chromatographic fingerprint of Rhizoma Coptidis extract**. Number shows in the bracket(s) represent the relative retention of the peak to the marker peak: 1 (0.81); 2 (0.88); 3 (0.89, jatrorrhizine); 4 (0.90); 5 (0.93, coptisine); 6 (0.98, palmatine); 7 (marker, berberine).

Each herb possesses a unique chemical profile of secondary metabolites which may be used as marker compounds for identification and standardization purposes. Some of these marker compounds have been related to the therapeutic efficacy of the herbs, as exemplified by our recent discovery of magnolol and honokiol as the active antispasmodic effects of *Cortex Magnolia Officinalis *[16]. Therefore, the marker content, especially that of biologically active compounds, may be used to confirm both the identity and quality of a herb. Additional File 1 summarizes the status of the qualitative (fingerprinting) and quantitative (HPLC) analyses of the herbs. Figure 3 shows the HPLC fingerprint of *Rhizoma Coptidis *as an example, whereas Table 3 provides the quantitative results of individual herbs. For those herbs that have CP and/or HKCMMS limits for the markers, the marker contents were found to be above the limits in all cases.

**Table 3:** Quantitative assay results of the component herbs

Pharmaceutical name	Reference marker	Limit (minimum)	Analytical results
*Radix Angelicae Dahuricae*	Imperatorin	0.080%^a^	0.081%
*Herba Artemisiae Scopariae*	Chlorogenic acid	--	0.31%
*Rhizoma Atractylodis Macrocephalae*	--	--	--
*Radix Aucklandiae*	Sum of costunolide and dehydrocostus lactone	2.2%^b^	2.7%
*Radix Bupleuri*	Saikosaponin a	0.16%^b^	0.43%
*Pericarpium Citri Reticulatae*	Hesperidin	3.5%^a^	6.5%
*Radix Codonopsis*	Lobetyolin	0.029%^b^	0.069%
*Semen Coicis*	Glycerol trioleate	0.50^a^	1.1%
*Rhizoma Coptidis*	Berberine Palmatine	4.1%^b^ 0.30%^b^	7.4% 1.8%
*Cortex Fraxini*	Sum of aesculetin and esculin	1.0%^a^	2.1%
*Radix et Rhizoma Glycyrrhizae Praeparata cum Melle*	Glycyrrhizic acid	--	2.7%
*Cortex Magnoliae Officinalis*	Sum of magnolol and honokiol	2.0%^b^	2.3%
*Radix Paeoniae Alba*	Paeoniflorin	1.6%^a^	1.8%
*Semen Plantaginis*	--	--	--
*Cortex Phellodendri Amurensis*	Berberine Palmatine	0.33%^b^ 0.18%^b^	0.95% 0.40
*Herba Pogostemonis*	Patchouli alcohol	0.10%^a^	0.23%
*Poria*	--	--	--
*Radix Saposhnikoviae*	Sum of prim-*O*-Glucosylcimifugin and 5-*O*-methylvisammioside	0.24%^b^	0.42%
*Fructus Schisandrae Chinensis*	Schisandrin	0.40%^a^	0.66%
*Rhizoma Zingiberis Praeparatum*	6-Gingerol	--	0.31%

^a ^Limit required by the Pharmacopoeia of the People's Republic of China (2005 edition).

^b ^Limit required by the Hong Kong Chinese Materia Medica Standards.

Inter-laboratory validation of fingerprint and quantitative HPLC demonstrated that the absolute deviation from mean (ADM) values of honokiol and magnolol in *Cortex Magnoliae Officinalis *were 0.43 and 1.11% respectively, confirming method reproducibility of the present study (Table 4). Similar results were obtained for all other herbs and no significant discrepancies were noted among the findings in Hong Kong and Australian laboratories.

**Table 4:** Inter-laboratory validation of quantitative assay of *Cortex Magnoliae Officinalis*

	Percentage content (%)		ADM (%)
	CUHK result	UWS result	
Honokiol	0.8355	0.8427	0.43
Magnolol	1.4815	1.5148	1.11

Note: ADM (absolute deviation from mean) = (| D1 - mean|/mean) × 100%, where mean = (D1+D2)/2; D1 = the first value, D2 = the second value

CUHK: Chinese University of Hong Kong

UWS: University of Western Sydney

**Figure 4:**
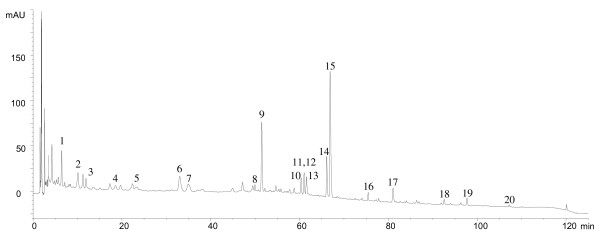
**Chromatographic fingerprint of the IBS-20 formula**.

The chemical standardization of the IBS-20 formula employed a HPLC-DAD-APCIMS system (Figure 4). Mass spectral analysis revealed 20 marker compounds attributable to ten herbs, of which eight markers were sufficient for quantitative estimation (Table 5). The fact that not all markers of the 20 herbs were detected was most likely due to low solubility of the lipophilic markers in the aqueous decoction. For the three herbs that have no established markers, namely, *Rhizoma Atractylodis Macrocephalae*, *Semen Plantaginis*, and *Poria*, no attempt was made to identify any ingredient from them.

**Table 5:** Identification of markers in the HPLC fingerprint of the formula by LC-MS analysis

			APCI MS Data (Positive Ion)				
					
Peak	Identification	Time (min)	M/QMIP^a^	Other Peaks	MS^2 ^of M/QMIP	Plant Source^b^	Content in formulation (mg/kg)
1	Esculin	6.4	341 (M+H)^+^	179	179	FR	
2	Chlorogenic acid	10.3	355 (M+H)^+^	163	163	AS	
3	Aesculetin	12.0	179 (M+H)^+^		134, 123, 109	FR	310
4	Paeoniflorin	18.8	498 (M+H_2_O)^+^	301, 179	301, 179	PL	
5	prim-*O*-Glucosylcimifugin	23.8	469 (M+H)^+^		307	SD	
6	Magnoflorine	32.2	342 (M)^+^		297, 265	CC, PA	
7	Liquiritin	35.9	419 (M+H)^+^	307, 257		GU	
8	5-*O*-Methylvisamminoside	49.9	453 (M+H)^+^	291	290	SD	
9	Hesperidin	52.0	610 (M)^+^	465, 449, 303	463	CR	1460
10	Columbamine	60.2	338 (M)^+^		323, 294	CC	
11	Jatrorrhizine	61.3	338 (M)^+^		323, 294	CC	
12	Epiberberine	61.3	336 (M)^+^			CC	
13	Coptisine	61.4	320 (M)^+^		304, 292	CC	
14	Palmatine	66.5	352 (M)^+^		337, 308	CC, PA	420
15	Berberine	67.3	336 (M)^+^		321, 292	CC, PA	1620
16	Glycyrrhizic acid	76.2	823 (M+H)^+^	647, 471, 453, 406		GU	780
17	Schisandrin	81.8	433 (M+H)^+^	415		SC	140
18	Honokiol	93.3	266 (M)^+^	263		MO	63
19	Magnolol	98.6	266 (M)^+^	261		MO	93
20	Schisandrin A	107.7	417 (M+H)^+^		402, 347, 316	SC	

Note: Peak number refers to the chromatographic fingerprint of the clinical preparation (Figure 4). Peaks 6, 10, 12, and13 were identified by LC-MS/MS2 analysis based on the literature values [17,18] and other peaks were identified by comparison of authentic chemicals. Jatrorrhizine and epiberberine (peaks 11, 12) were co-eluted with the same retention times.

^a ^Molecular or quasi-molecular ion peak; ^b ^FR: *Fraxinus rhynchophylla*; AS: *Artemisia scoparia*; PL: *Paeonia lactiflora*; SD: *Saposhinikovia divaricata*; GU: *Glycyrrhiza uralensis*; CR: *Citrus reticulata*; CC: *Coptis chinensis*; PA: *Phellodendron amurense*; SC: *Schisandra chinensis*; MO: *Magnolia officinalis*.

**Figure 5:**
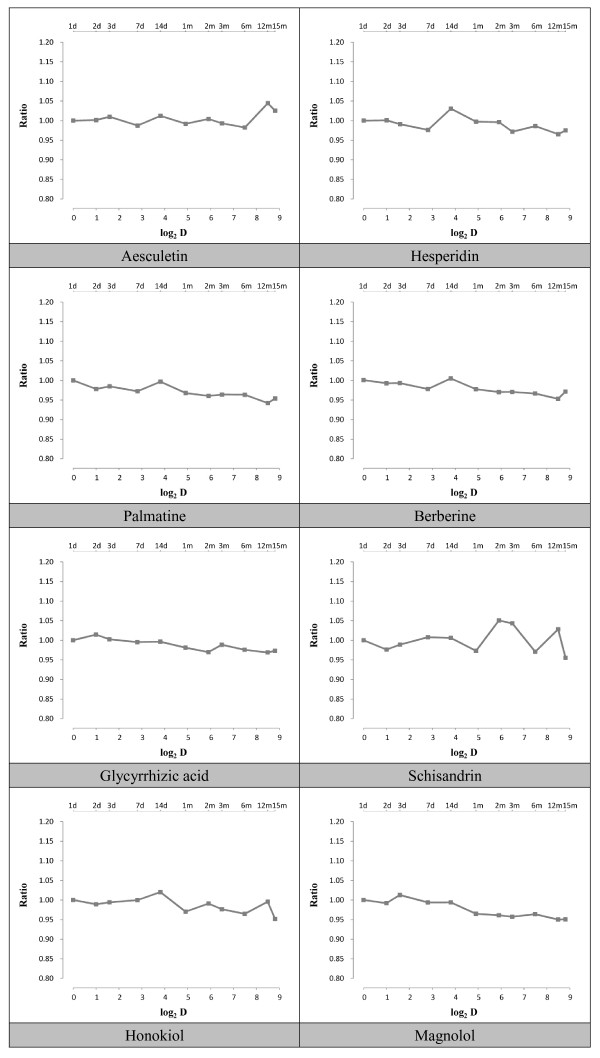
**Stability of the IBS-20 formula**. The ratio was calculated by the content of the marker at the date of measurement to that at the starting date.

The stability results showed that the concentrations of these compounds did not change significantly from the date of production (day 0) to the last day of analysis, confirming the chemical stability of the IBS-20 formula under the storage conditions (Figure 5).

## Conclusion

A QA/QC program involving good supply practice acquisition, botanical validation, chemical profiling of the component herbs, as well as the establishment of a chemical standardization protocol and stability monitoring has been implemented on a 20-herb botanical preparation, the IBS-20 formula. The results of this study demonstrate that it is possible to establish a QA/QC program to monitor the quality of poly-herbal formulations employing botanical and chemical methods. In particular, the generation of a fingerprint HPLC chromatographic protocol in which the identities of a series of appropriate marker compounds, including relevant biologically active constituents, were identified for use in product standardization, coupled with a stability study procedure involving the LC-MS quantitation of major chemical markers, represent major advances in the development of quality control methods for poly-herbal Chinese medicine products for clinical studies and therapy.

## Abbreviations

ADM: absolute deviation from mean; APCIMS: atmospheric pressure chemical ionization mass spectrometry; CP: Pharmacopoeia of the People's Republic of China; DAD: diode array detection; GMP: good manufacturing practice; HKCMMS: Hong Kong Chinese Materia Medica Standard; HPLC: high-performance liquid chromatography; IBS: irritable bowel syndrome; ICP-MS: inductively coupled plasma-mass spectrometry; GS: gas chromatography; LC-MS: liquid chromatography-mass spectrometry; NMR: nuclear magnetic resonance; QA: quality assurance; QC: quality control; TLC: thin-layer chromatographic; UV: ultra violet; WHO: World Health Organization.

## Competing interests

The authors declare that they have no competing interests.

## Authors' contributions

SPI coordinated the research and drafted the manuscript. MZ, YFX, MLC, YYZ, YWT and SHT performed the experiments. JJYS, AB, BB, HHSF and CTC supervised this project and revised the manuscript. All authors read and approved the final version of the manuscript.

## Supplementary Material

Additional file 1 Summary of the herbs and their chemical marker. Click here for file
