# Supplementary material for: Quality assurance for Chinese herbal formulae: standardization of IBS-20, a 20-herb preparation
Source: Chin Med. 2010 Feb 22;5:8. doi: 10.1186/1749-8546-5-8 (PMC2845568; doi:10.1186/1749-8546-5-8)
Supplement: Additional file 1 — Summary of the herbs and their chemical marker. [file 1749-8546-5-8-S1.DOC]

**Additional file 1** Summary of the herbs and their chemical markers

| **Pharmaceutical names** | **Chinese names** | **Botanical names** | **Plant part** | **Qualitative analysis**  **(Fingerprinting)** | | **Quantitative analysis** | |
| --- | --- | --- | --- | --- | --- | --- | --- |
|  |  |  |  | **Reference marker** | **Method** | **Reference marker** | **Method** |
| *Radix Angelicae Dahuricae* | *Baizhi* | *Angelica dahurica* (Fisch.ex Hoff.) Benth. et Hook. f. (Apiaceae) | Root | Imperatorin | HPLC ^b^ | Imperatorin | HPLC |
| *Herba Artemisiae Scopariae* | *Yinchen* | *Artemisia scoparia* Waldst. et Kit. (Asteraceae) | Aerial part | Chlorogenic acid | HPLC ^b^ | Chlorogenic acid | HPLC |
| *Rhizoma Atractylodis Macrocephalae* | *Baizhu* | *Atractylodes macrocephala* Koldz．(Asteraceae) | Rhizome | -- | HPLC ^b^ | -- | -- |
| *Radix Aucklandiae* | *Muxiang* | *Aucklandia lappa* Decne. (Asteraceae) | Root | Costunolide | HPLC ^a^ | Costunolide,  Dehydrocostus lactone | HPLC |
| *Radix Bupleuri* | *Chaihu* | *Bupleurum chinense* DC. (Apiaceae) | Root | Saikosaponins a and d | HPLC ^a^ | Saikosaponin a | HPLC |
| *Pericarpium Citri Reticulatae* | *Chenpi* | *Citrus reticulata* Blanco (Rutaceae) | Ripe fruit pericarp | Hesperidin | HPLC ^b^ | Hesperidin | HPLC |
| *Radix Codonopsis* | *Dangshen* | *Codonopsis pilosula* Nannf．var．modesta (Nannf.) L T. Shen (Campanulaceae ) | Root | Lobetyolin | HPLC ^a^ | Lobetyolin | HPLC |
| *Semen Coicis* | *Yiyiren* | *Coix lacryma-jobi* L.var.*ma-yuen* (Roman.) Stapf (Poaceae) | Ripe kernel | Glycerol trioleate | HPLC ^b^ | Glycerol trioleate | HPLC |
| *Rhizoma Coptidis* | *Huanglian* | *Coptis chinensis* Franch.(Ranunculaceae) | Rhizome | Berberine | HPLC ^a^ | Berberine  Palmatine | HPLC |
| *Cortex Fraxini* | *Qinpi* | *Fraxinus rhynchophylla* Hance (Oleaceae) | Branch or stem bark | Aesculetin | HPLC ^b^ | Aesculetin  Esculin | HPLC |
| *Radix et Rhizoma Glycyrrhizae Praeparata cum Melle* | *Zhigancao* | *Glycyrrhiz uralensis Fisch.*(Fabaceae) | Root and rhizome | Glycyrrhizic acid | HPLC ^b^ | Glycyrrhizic acid | HPLC |
| *Cortex Magnoliae Officinalis* | *Houpo* | *Magnolia officinalis* Rehd. et Wils．(Magnoliaceae) | Root, branch, stem bark | Magnolol | HPLC ^a^ | Magnolol  Honokiol | HPLC |
| *Radix Paeoniae Alba* | *Baishao* | *Paeonia lactiflora* Pall. (Paeoniaceae) | Root | Paeoniflorin | HPLC ^a^ | Paeoniflorin | HPLC |
| *Semen Plantaginis* | *Cheqianzi* | *Plantago asiatica* L. (Plantaginaceae) | Ripe seed | -- | HPLC ^b^ | -- | -- |
| *Cortex Phellodendri Amurensis* | *Guanhuangbo* | *Phellodendron amurense* Rupr. (Rutaceae) | Bark | Berberine | HPLC ^a^ | Berberine  Palmatine | HPLC |
| *Herba Pogostemonis* | *Guanghuoxiang* | *Pogostemon cablin* (Blanco) Benth.( Lamiaceae) | Aerial part | -- | HPLC ^b^ | Patchouli alcohol | GC |
| *Poria* | *Fuling* | *Poria cocos* (Schw.) Wolf (Polyporaceae) | Sclerotium | -- | HPLC ^b^ | -- | -- |
| *Radix Saposhnikoviae* | *Fang Feng* | *Saposhnikovia divaricata* (Turcz.) Schischk. (Apiaceae) | Root | 5-*O*-Methyl- visammioside | HPLC ^a^ | prim-*O*-Glucosylcimifugin, 5-*O*-Methylvisammioside | HPLC |
| *Fructus Schisandrae Chinensis* | *Wu Wei Zi* | *Schisandra chinensis* (Turcz.)Baill.( Schisandraceae) | Ripe fruit | Schisandrin | HPLC ^b^ | Schisandrin | HPLC |
| *Rhizoma Zingiberis Praeparatum* | *Pao Jiang* | *Zingiber officinale* Rose (Zingiberaceae) | Prepared rhizome | 6-Gingerol | HPLC ^b^ | 6-Gingerol | HPLC |

^a^ Hong Kong Chinese Materia Medica Standards

^b^ The HPLC fingerprint was established and compared with authentic sample by in-house method. Analytical procedures for individual herbs are available from the corresponding author upon request.
